# Use of selected complementary and alternative medicine (CAM) treatments in veterans with cancer or chronic pain: a cross-sectional survey

**DOI:** 10.1186/1472-6882-6-34

**Published:** 2006-10-06

**Authors:** F Patricia McEachrane-Gross, Jane M Liebschutz, Dan Berlowitz

**Affiliations:** 1 Edith Nourse Rogers Memorial Veterans Hospital, 200 Springs Road, Bedford, MA, USA; 2 Martin Army Community Hospital, Building 9200, Fort Benning, GA, USA; 3 Section of General Internal Medicine, Boston University School of Medicine, Boston Medical Center, USA

## Abstract

**Background:**

Complementary and alternative medicine (CAM) is emerging as an important form of care in the United States. We sought to measure the prevalence of selected CAM use among veterans attending oncology and chronic pain clinics and to describe the characteristics of CAM use in this population.

**Methods:**

The self-administered, mail-in survey included questions on demographics, health beliefs, medical problems and 6 common CAM treatments (herbs, dietary supplements, chiropractic care, massage therapy, acupuncture and homeopathy) use. We used the chi-square test to examine bivariate associations between our predictor variables and CAM use.

**Results:**

Seventy-two patients (27.3%) reported CAM use within the past 12 months. CAM use was associated with more education (p = 0.02), higher income (p = 0.006), non-VA insurance (p = 0.003), additional care outside the VA (p = 0.01) and the belief that lifestyle contributes to illness (p = 0.015). The diagnosis of chronic pain versus cancer was not associated with differential CAM use (p = 0.15). Seventy-six percent of CAM non-users reported that they would use it if offered at the VA.

**Conclusion:**

Use of 6 common CAM treatments among these veterans is lower than among the general population, but still substantial. A large majority of veterans reported interest in using CAM modalities if they were offered at the VA. A national assessment of veteran interest in CAM may assist VA leaders to respond to patients' needs.

## Background

Eisenberg and colleagues estimated that Americans made approximately 425 million visits to CAM practitioners in 1990, more than the total number of visits to primary care physicians during that period [1]. A large-scale follow-up study showed that CAM use among the general public increased from 34% in 1990 to 42% in 1997 [2]. By 2001, hospitals that offered CAM services were citing patient demand as a primary motivating factor [3]. CAM is emerging as an important form of care.

The National Center for Complementary and Alternative Medicine (NCCAM) defines Complementary and Alternative Medicine (CAM) as "a group of diverse medical and health care systems, practices, and products that are not presently considered to be part of conventional medicine" [4]. This definition, arrived at after much debate [5], demonstrates that the scope of CAM can be quite large and dynamic. The American Cancer Society separately defines "complementary" and "alternative." "Complementary" methods are supportive methods used to complement evidence-based treatment. Complementary therapies do not replace mainstream cancer treatment and are not promoted to cure disease. Rather, they control symptoms and improve well-being and quality of life. Alternative methods are defined as unproved or disproved methods, rather than evidence-based or proven methods to prevent, diagnose, and treat cancer [6].

Within the epidemiological literature on CAM use, variations in the definition of CAM, study populations and methodologies make it very difficult to compare studies and reach firm conclusions. These difficulties notwithstanding, it is apparent that CAM use continues to be significant. A survey developed by NCCAM and the National Center for Health Statistics (NCHS) conducted in 2002 revealed that 36% of US adults had used some form of CAM in the year prior to the survey. When prayer for health-related purposes was included in the definition of CAM, the prevalence rose to 62% [6].

Some experts suggest that CAM use is predictably increased in situations such as cancer, where "illness consequences are high and beliefs in the effectiveness of conventional treatments (i.e. response efficacy) are low" [7]. Indeed, several studies have shown that cancer patients are increasingly incorporating complementary therapies into their overall treatment [8,9]. In one systematic review of the use of CAM among patients with cancer, the reported prevalence rate ranged from 7 to 64% [10]. In 2001, an update from NCCAM pointed out that among cancer patients, the use of CAM ranges between 30 and 75% worldwide [12]. Even higher rates, ranging from 67% to 83% have been documented in patients with breast cancer [11,13]. At a comprehensive cancer center, when CAM was broadly defined to include spiritual practices and psychotherapy, 83.3% of subjects had used at least one CAM approach [10].

Chronic pain is also an area where CAM use has flourished. In 1997, researchers found that 54% of Americans who reported back or neck pain in the previous 12 months had used complementary therapies to treat their condition. At that time, chiropractic therapy was the most common "unconventional" therapy used in the United Sates [11]. The more recent NCCAM/NCHS survey [6] supports these findings from 1997, indicating that CAM approaches are very often used to treat body pain, especially in the back and neck. This survey also revealed CAM use is greater among certain groups such as women, those with higher education, those who had been hospitalized within the previous year, and former smokers.

In keeping with the trend toward increased support of CAM use, the Department of Veterans Affairs Health Care Programs Enhancement Act of 2001 required the Veterans Administration (VA) Health Care System to provide chiropractic programs nationwide [12]. However, little is known about the use of CAM or patient demand for such services within the VA, which manages the largest integrated healthcare system in the US.

Data on CAM use in veterans is limited to one geographic location in Southern Arizona where CAM use may be influenced by a high level of advertisement and promotion [13,14]. In this location, 49.6% of veterans reported current or previous use of CAM.

The primary purpose of this study was to ascertain the prevalence of 6 common CAM treatments and determine characteristics associated with their use among veterans whose CAM use was expected to be high, those receiving care at the oncology and chronic pain clinics [8]. We hypothesized that CAM use would be lower than that of the general population and would be influenced by income, educational level, insurance status, diagnosis [2,8,20] and beliefs about the cause of illness [21,22]. National surveys show women are more likely to utilize CAM than men [1]; as such, we expected to find low prevalence of CAM use among our predominantly male veteran population. We also hypothesized that CAM use among veterans who received additional care outside the VA would be higher than use among veterans who received care only at the VA because seeking care outside the VA is correlated with higher income[15,16]

## Methods

### Site and participant recruitment

The study was conducted through the outpatient oncology and pain clinics at the Jamaica Plain campus of the VA Boston Health Care System (VABHCS), which serves as the major tertiary and surgical referral center for all other VA facilities in New England. To minimize sampling bias, every 5^th ^name was selected from both alphabetical databases of individuals who had attended the clinics at least once during the 12 months prior to the study start date of April 1, 2003. Medical directors of each clinic generated letters to subjects informing them of study requirements and giving them the assurance of confidentiality. The Institutional Review Boards at VABHCS and Edith Nourse Rogers Memorial Veterans Hospital (Bedford, MA) approved the study.

We used a modified Dillman protocol [17] to recruit participants to complete a mail-in survey. Two weeks after sending out a letter of invitation to participate from the medical director of each clinic, we performed the first mailing of the survey and included a $5 cash incentive along with an addressed, stamped envelope. Two weeks after the first mailing, we sent a reminder postcard to those who had not yet responded and two weeks after that, we performed the final mailing of the survey. Participants mailed back a separate postcard with their identifying information to keep track of responses while maintaining anonymity of the survey. We used a conservative estimation of prevalence of CAM use (20%) to obtain our sample size calculation of 264.

### Survey instrument

We developed a 44-question anonymous instrument that was reviewed by a panel of physicians and pilot tested by a group of veterans. Our pilot test subjects completed the survey in ≤ 30 minutes. Four domains were covered: demographics including military service, health status and beliefs about the cause of health problems, medical problems and treatments, and use of CAM. Demographic information included age, gender, race/ethnicity, religion, education, employment status, income, and health insurance status. A question on beliefs about contributing factors to health problems was included.

A participant was considered to have been a CAM user if s/he reported use of one or more of the following modalities in the previous 12 months: herbs, dietary supplements, chiropractic care, massage therapy, acupuncture and homeopathy. We chose CAM modalities most likely to be familiar to the largest number of veterans [1,18,19] and for the sake of clarity, excluded modalities such as vitamin therapy which may be prescribed as part of a conventional regime (such as vitamins B12 and E). We asked participants to identify what medical problems they had, place and type of treatment, assessment of usefulness of treatments as well as source of information about medical treatments. The words "alternative," "complementary" or "unconventional" were not included in the survey and CAM choices were integrated among conventional modalities in the questions about medical treatments used. Participants who reported CAM use were asked about duration of use, expected benefits, current satisfaction with these treatments, discussion with VA provider and money spent on CAM per month. All participants were asked about preferences regarding CAM use if offered at the VA.

### Statistical analysis

In this paper, we present descriptive data on respondent characteristics, CAM modalities used, duration and satisfaction of use. Based on the existing literature [1,3] and current theories, we predicted that education, income, insurance status, additional non-VA healthcare, and beliefs about the cause of illness would be associated with CAM use. We used the chi-square test to examine bivariate associations between our predictor variables and CAM use. All analyses were performed with PC-SAS, version 8 for Windows (SAS Institute, Inc., Cary, North Carolina).

## Results

### Characteristics of survey respondents

Surveys were mailed to 500 veterans; 457 were alive with correct mailing addresses (39 patients from oncology and 4 from the chronic pain clinic were deceased). We received 264 responses (57.8%), with no significant difference between the oncology clinic patients (55.9%) and chronic pain clinic patients (59.3%). The mean age was 65 years, 93.5% were male and 91.6% were white. The majority was unemployed, unable to work or retired (82.4%), reported an income of ≤ $50 K (97.2%), and had no additional insurance apart from their VA benefits (54.1%). See Table 1. We were not able to compare characteristics of respondents with non-respondents. However, the data do compare to demographic profiles of veterans receiving ambulatory care in the Boston, MA area [20]. Back pain was the most frequently reported medical problem (62.5%) and most veterans (76.9%) had used prescription pills to treat their medical problems. A large majority (78%) identified their VA provider as the main source of information about their medical problems.

**Table 1:** Selected characteristics of survey respondents

**Characteristic**	**Total Sample** n = 264 N(%) or mean (SD)	**CAM users** n = 72 N(%) or mean (SD)	**CAM non-users** n = 192 N(%) or mean (SD)	**P value**
Mean age (SD) in years	65.0 (13.9)	63.1 (14.1)	65.7 (13.8)	0.82
Gender				
Male	245 (93.5)	67 (93.1)	178 (93.7)	0.85
Female	17 (6.5)	5 (6.9)	12 (6.3)	
Race/Ethnicity				
White non-Hispanic	241 (91.6)	66(91.7)	175 (91.6)	0.99
Other	22 (8.4)	6 (8.3)	16 (8.4)	
Religion				
Catholic	150 (57.0)	41 (57.0)	109 (57.1)	0.77
Protestant	64 (24.3)	19 (26.4)	45 (23.6)	
Other	49 (18.6)	12 (16.6)	37 (19.3)	
Employment status				
Employed (full or part-time, homemaker)	46 (17.6)	19 (26.4)	27 (14.3)	0.21
Unemployed or unable to work	102 (39.1)	24 (33.3)	78 (41.3)	
Retired	113 (43.3)	29 (40.3)	84 (44.4)	
Income				
≤$50 K	243 (97.2)	62 (91.2)	181 (99.4)	0.006
>$50 K	7 (2.8)	6 (8.8)	1 (0.6)	
Education				
<High School or GED	94 (36.2)	19 (26.4)	75 (39.9)	0.02
≥High School or GED	166 (63.8)	53 (73.6)	113((60.1)	
Insurance status				
Additional insurance	111 (45.9)	43 (60.6)	68 (39.8)	0.003
No additional insurance	131 (54.1)	28 (39.4)	103 (60.2)	
Care outside VA				
Additional care	54 (20.5)	22 (30.6)	32 (16.7)	0.01
No additional care	210 (79.5)	50 (69.4)	160 (83.3)	
Beliefs about cause of illness				
Lifestyle contributes to illness	144 (54.5)	48 (66.7)	96 (50.0)	0.001
Lifestyle does not contribute to illness	120 (45.5)	24 (33.7)	96 (50.0)	
Referring Clinic Sample				
Pain Clinic	118 (44.7)	27 (37.5)	91(47.4)	0.15
Cancer Clinic	146 (55.3)	45 (62.5)	101(52.6)	

Percentages are based on actual numbers of persons reporting data for each item (ranges from 242–264)

### CAM users

CAM use in this population was 27.3%. Out of the 6 CAM modalities presented to respondents in this survey (Table 2), dietary supplements were the most frequently used (51.4%). The majority (63.9%) had been using CAM for more than 2 years and 35% were satisfied with these treatments. Most (89%) of our CAM users had discussed their use of CAM with their VA provider. Eighteen percent reported using CAM instead of prescribed treatments. Forty-one percent of CAM users in this population reported spending ≥ $50/month on these modalities.

**Table 2:** Selected Characteristics among CAM users (n = 72)

	**N (% of CAM users)**
*Modalities (n = 72)	
Dietary supplements	37 (51.4)
Massage therapy	22 (30.6)
Chiropractic care	19 (26.4)
Herbs	19 (26.4)
Acupuncture	7 (9.7)
Homeopathy	3 (4.2)
Other	4 (5.6)
*Duration of use (n = 61)	
More than 2 years ago	39 (63.9)
Within the last 2 years	22 (36.1)
*Satisfaction with treatments (n = 65)	
Yes	23 (35.4)
No	17 (26.2)
Somewhat	25 (38.4)
Use of CAM instead of prescribed treatments (n = 60)	
Yes	11 (18.3)
No	49 (81.7)
*Discussed use of CAM with VA provider (n = 64)	
Yes	57 (89.1)
No	7 (11.9)
*Money spent on CAM per month (n = 64)	
Do not spend any money	13 (20.3)
<$50	19 (29.7)
$50–$75	7 (10.9)
$76–100	5 (7.8)
>$100	14 (21.9)
Do not know	6 (9.4)

*Percentages are based on actual numbers of persons reporting data for each item

### Characteristics associated with CAM use

CAM use was associated with higher socioeconomic status and having insurance in addition to VA benefits (Table 1). CAM users were also more likely to be receiving additional care outside the VA than CAM non-users (30.6% vs. 16.7%; p = 0.01). Two-thirds (66.7%) of CAM users believed that their lifestyle contributed to their health problems, as compared to 50% of CAM non-users (p = 0.015). CAM use did not differ between pain clinic subjects and oncology clinic subjects (30.8% vs. 22.9%; p = 0.15). A higher percentage of patients getting additional care outside the VA used CAM in comparison to those who only received VA care (40.7% vs. 23.8%; p = 0.01). A large majority of non-CAM users (76%) reported they would use CAM modalities if they were offered at the VA.

## Discussion

We undertook this study to ascertain the prevalence and characteristics of use of 6 common CAM treatments among a group of veterans who were receiving outpatient care for cancer or chronic pain within a VA Health Care System. Twenty seven percent of veterans reported use of these complementary and alternative medicine treatments in the prior 12 months. While this is a substantial number, it is lower than what has been reported for the general population. This may be due in part to methodological differences among CAM studies but is consistent with previous findings of lower prevalence of CAM use among males compared to women[1,21] It should be noted however, that although the gender difference is clear, there are studies that have demonstrated a relatively high prevalence of CAM use (>40%) among older men with cancer[22,23] and chronically painful conditions[24]

Variations in prevalence of CAM use may also reflect differences in geographic location. As noted earlier, the only other study assessing veteran use of CAM was located in the southwest United States where use of CAM is more common [14]. When compared to this group in Southern Arizona, with similar age and gender characteristics (mean age of CAM users = 61.9 years; mean age of non-CAM users = 62.7 years, CAM users = 90.5% men, non-CAM users = 93.4% men), reported CAM use in our group was in fact lower (27% vs. 49.6%).

Another potential reason for overall lower use of CAM in our study is the group's low income. Only 19% reported annual incomes above $30,000, similar to findings in other studies [2,3,19]. CAM use in this population was associated with higher socioeconomic status and having insurance. A large majority of CAM non-users in this population (76%) did report that they would use these modalities if they were offered within the VA healthcare system. It is possible that increased health insurance coverage of well-studied CAM therapies may lead to increased use of these therapies.

The definition of CAM has not been uniform across the many studies assessing prevalence of CAM use. For example, in Eisenberg's landmark telephone survey [1], the list of "unconventional therapies" used by 34% of Americans included spiritual healing, commercial weight-loss programs, lifestyle diets and self-help groups, although users of these constituted a small minority. A 2002 NHIS survey found that when excluding prayer, meditation and relaxation was the second most common CAM treatment [6]. In the Southern Arizona study, subjects were asked whether they "currently use or have\ldotsever used complementary and alternative medicine." Only if subjects asked for clarification of CAM were they given examples from the categories outlined by NCCAM [4]. In a follow-up qualitative study of 100 of those CAM users [14], the researchers do report that those subjects were using a wide range of CAM modalities, not including alternative diets. In our study, we chose CAM modalities most likely to be familiar to the largest number of veterans [1,22,24] and for the sake of clarity, excluded modalities such as vitamin therapy which may be prescribed as part of a conventional regime (examples: vitamins B12 and E). We also excluded prayer, and when compared to the most recent national, comprehensive survey [6], the use of CAM in our more local study was slightly lower (27.3% versus 36%). In the national study, when prayer specifically for health reasons was included in the definition of CAM, use increased to 62%. Uniformity of CAM definition is likely to increase as researchers become more familiar with NCCAM categories.

There seems to be a lingering perception in the literature that individuals who use non-conventional treatments for their medical problems reject conventional care [25]. This study supports the idea that most CAM users employ these modalities in conjunction with conventional medicine [2,6,26,27] by affirming that there are CAM users among those who seek treatment in conventional medical settings. Because the study only sampled users of conventional medicine, it was unable to test the hypothesis that CAM users may reject conventional care. Unlike other studies, however, a very high percentage of CAM users in this study reported discussing their use of CAM with their VA providers [26,28,29]. One explanation may be that users of veterans administration health services have a high level of satisfaction with their health care providers[30]. Future studies might address the question of satisfaction in the provider and disclosure of CAM treatment. Another possible explanation for this finding might be the change in public opinion about CAM and presumption among patients that this is a legitimate aspect of treatment to discuss with their provider. In the 1998 survey conducted by Eisenberg et al, 70% of respondents reported they sought care from both conventional and alternative providers at the same time [31]. The recent NCCAM/NCHS survey [6] seems to support this view. In that study, 25.8% of adults who used CAM during the past 12 months did so because a conventional health care provider suggested it. Furthermore, these patients were all followed in specialty clinics in which the clinicians may be attuned to potential use of CAM among their patients. This would need to be reevaluated among veterans using non-specialty services.

One major limitation of this study is its cross-sectional design. Longitudinal studies could track changes in the use of CAM over time, especially if these therapies were introduced to the VA healthcare system. Another major limitation is the potential underestimation of CAM use by including only 6 therapies. However, these are the CAM modalities most likely to be known by the majority of veterans. Relationships between respondent characteristics and the CAM modalities in this study likely reflect those of all CAM modalities. Length of time since cancer diagnosis could influence use of CAM but this was not asked of respondents. However, the limited information about this in the medical literature suggests that this is not a strong predictor of CAM use [32]. This has not been studied among veterans. Further studies on the topic should include all users of VA services.

Lastly, we do not know the characteristics of the survey non-respondents, but the demographic characteristics of our study subjects reflect those of the VA population served at the medical center [25].

## Conclusion

In spite of its limitations, our study provides valuable information about the use of CAM among veterans. Prevalence of CAM use and desire for CAM availability was higher than we expected. Our findings confirm those of an earlier study conducted in a geographic location where CAM use is known to be prevalent. Especially important is the finding that a large majority of veterans who do not use CAM state they would do so if these therapies were available within the VA system. Future research should investigate this assertion and its potential cost/benefit implications. This information would be helpful to healthcare and insurance decision-makers as they develop future policies and services. Our data also adds to the increasing body of evidence that there is substantial CAM use among US adults and that CAM users do not reject conventional medicine. Providers caring for veterans should therefore be proactive in communicating with patients about their use of CAM, seeking to identify their needs and involve them in the treatment decision-making process.

## Competing interests

The author(s) declare that they have no competing interests.

## Authors' contributions

FPM-G conceived of the study, developed the survey, coordinated data collection, analyzed the data and drafted the manuscript, DB participated in the study design, statistical analysis and manuscript editing; JML obtained grant funding for the study, participated in study design, survey development, data analysis and manuscript editing.

## Pre-publication history

The pre-publication history for this paper can be accessed here:


